# Alpha reactivity to first names differs in subjects with high and low dream recall frequency

**DOI:** 10.3389/fpsyg.2013.00419

**Published:** 2013-08-13

**Authors:** Perrine Ruby, Camille Blochet, Jean-Baptiste Eichenlaub, Olivier Bertrand, Dominique Morlet, Aurélie Bidet-Caulet

**Affiliations:** ^1^Brain Dynamics and Cognition Team, Lyon Neuroscience Research Center, INSERM, CNRSLyon, France; ^2^University Lyon 1Lyon, France

**Keywords:** dreaming, sleep, self, 8–12 Hz, consciousness, inhibition, oddball, novelty

## Abstract

Studies in cognitive psychology showed that personality (openness to experience, thin boundaries, absorption), creativity, nocturnal awakenings, and attitude toward dreams are significantly related to dream recall frequency (DRF). These results suggest the possibility of neurophysiological trait differences between subjects with high and low DRF. To test this hypothesis we compared sleep characteristics and alpha reactivity to sounds in subjects with high and low DRF using polysomnographic recordings and electroencephalography (EEG). We acquired EEG from 21 channels in 36 healthy subjects while they were presented with a passive auditory oddball paradigm (frequent standard tones, rare deviant tones and very rare first names) during wakefulness and sleep (intensity, 50 dB above the subject's hearing level). Subjects were selected as High-recallers (HR, DRF = 4.42 ± 0.25 SEM, dream recalls per week) and Low-recallers (LR, DRF = 0.25 ± 0.02) using a questionnaire and an interview on sleep and dream habits. Despite the disturbing setup, the subjects' quality of sleep was generally preserved. First names induced a more sustained decrease in alpha activity in HR than in LR at Pz (1000–1200 ms) during wakefulness, but no group difference was found in REM sleep. The current dominant hypothesis proposes that alpha rhythms would be involved in the active inhibition of the brain regions not involved in the ongoing brain operation. According to this hypothesis, a more sustained alpha decrease in HR would reflect a longer release of inhibition, suggesting a deeper processing of complex sounds than in LR during wakefulness. A possibility to explain the absence of group difference during sleep is that increase in alpha power in HR may have resulted in awakenings. Our results support this hypothesis since HR experienced more intra sleep wakefulness than LR (30 ± 4 vs. 14 ± 4 min). As a whole our results support the hypothesis of neurophysiological trait differences in high and low-recallers.

## Introduction

Dreaming remains one of the great mysteries of human cognition. Just after waking up in the morning nearly everyone has experienced some bizarre representations from the night before. They are sometimes insightful and can result in great discoveries at the scientific level (like the periodic table or the ring-like structure of benzene, Maquet and Ruby, 2004) or at the personal level (Freud, 1899).

However, despite recent advances (Voss et al., 2009; Wamsley et al., 2010; Dresler et al., 2011, 2012; Marzano et al., 2011) dreaming remains a poorly understood cognitive ability (Maquet and Ruby, 2004; Hobson, 2005; Nielsen and Stenstrom, 2005). Indeed, very little is known about the psychological mechanisms constraining dream content and about the cerebral mechanisms involved in the production and encoding of the oneiric representations (Nir and Tononi, 2010; Ruby, 2011; De Gennaro et al., 2012; Perogamvros and Schwartz, 2012).

### Strategies classically used to investigate the cerebral correlates of dreaming and their limitations

In the 1950s, rapid eye movement sleep (REM sleep) was considered as the neurophysiological state underlying dreaming (Aserinsky and Kleitman, 1953; Dement and Kleitman, 1957a,b; Sastre and Jouvet, 1979). Following this hypothesis, some scientists restricted their investigation of the cerebral correlates of dreaming to the investigation of REM sleep using positron emission tomography (PET) (e.g., Maquet et al., 1996; Braun et al., 1998). However, the REM sleep hypothesis of dreaming has been challenged. First, according to several studies, REM sleep is not necessary for dreams to be reported. A significant amount of awakenings in non-REM (NREM) sleep are followed by a dream report (mean 43%, range 0–75%, for a review, see Nielsen, 2000), even if no REM sleep occurred before the NREM episode (e.g., Palagini et al., 2004; Noreika et al., 2009). Second, some studies have shown that REM sleep is not sufficient for dreams to be reported. Ten to Twenty percentage of awakenings from REM sleep are not followed by a dream report (Nielsen, 2000), and several neuropsychological studies have shown that lesions in the temporo-parietal junction (TPJ) and medial prefrontal cortex (MPFC) are associated with a cessation of dream reporting with no concomitant REM sleep disturbance (Murri et al., 1985; Solms, 1997, 2000; Bischof and Bassetti, 2004). These neuropsychological findings were a breakthrough in the research domain of sleep and dreaming. Indeed, based on these results, Solms (2000) argued that dreaming and REM sleep are dissociable states and that dreaming can occur in any sleep stage. According to this new hypothesis, investigating brain activity only during REM sleep is not sufficient to characterize the cerebral correlates of dreaming.

Interestingly, some researchers did investigate dreaming in several sleep stages. They used scalp electroencephalogram (EEG) and classified subject's awakenings (from REM and NREM sleep) according to the subsequent report (or not) of a dream. Then, they analyzed the EEG power in various frequency bands during both REM and NREM sleep in the few minutes preceding a dream report versus no dream report. They found that the sleep EEG preceding a dream report differed from the sleep EEG preceding no dream report (Takeuchi et al., 2003; Esposito et al., 2004; Chellappa et al., 2011; Marzano et al., 2011). However, the results proved to be heterogeneous and sometimes contradictory. For instance, after awakenings from NREM sleep, two studies found that a successful dream recall was associated with a decreased alpha power (8–12 Hz) in the sleep EEG preceding awakening (Esposito et al., 2004; Marzano et al., 2011), while another study found that successful dream recall was associated with increased alpha power (Takeuchi et al., 2003). The variability of these results (for a review see Chellappa and Cajochen, 2013) may be due to the difficulty in knowing for sure whether or not the reported dream occurred in the few minutes preceding awakening. Studies using such an approach may thus have been biased by dream reports resulting from dreams produced outside the few minutes of sleep investigated.

Other researchers exploited one of the only current means to obtain real time information about dreaming i.e., lucid dreaming. Lucid dreamers can be aware that they are dreaming while asleep and sometimes they can also be in control of some part/elements of the dreamed story. In that case a lucid dreamer can indicate to an experimenter that he is dreaming, for example by moving his eyes according to a particular sequence (a code) determined previously. Using this strategy researchers could contrast REM with lucid dreaming to REM without lucid dreaming and investigate the cerebral correlates of lucidity (Voss et al., 2009; Dresler et al., 2011, 2012). However, the great limitation of this approach is that lucid dreaming is (1) a particular type of dream (2) rare in the general population, rare in subjects who can experience it and even more rare during a night of sleep in a lab (Schredl and Erlacher, 2011). As a consequence, the few studies which investigated lucid dreaming obtained results about “lucidity” and not really dreaming and data from only one to three subjects.

Finally, in light of the literature, it appears that no convincing paradigms are available to investigate the brain activity associated with dreaming in healthy subjects. The main reason which explains this lack and the paucity of experimental results about the cerebral correlates of dreaming is the difficulty in knowing when dreaming occurs in the subjects sleep cycle. Indeed, in this context it is not possible to scan the brain while dreaming versus while not dreaming.

### A new approach to investigate the dreaming brain

Studies in cognitive psychology showed that personality (openness to experience, thin boundaries, absorption), creativity, nocturnal awakenings, and attitude toward dreams were significantly related to dream recall frequency (DRF) (Schredl et al., 2003). These results suggest the possibility of neurophysiological trait differences between subjects with high and low DRF. By testing this hypothesis, we chose a new approach to investigate dreaming (i.e., the investigation of dreaming-related cerebral traits) which avoids the methodological difficulties presented above. We compared the brain activity of healthy subjects with high and low DRF. By comparing these two groups (High recallers, HR; Low recallers, LR) during sleep and wakefulness, we investigate the cerebral organization promoting the dreaming process or the memorization of the dream content and possibly the cerebral mechanisms involved in dream production and/or memorization.

In a first study, we showed that event related potentials (ERPs) elicited by first names, presented rarely and randomly among pure tones (Eichenlaub et al., 2012) were dramatically different in HR (more than three dream recalls a week) and LR (less than two dream recalls a month) during both sleep and wakefulness (Eichenlaub et al., 2013). Responses to first names during wakefulness and N2 were larger in HR than in LR around 250 ms i.e., at the latency of the brain orienting response (novelty P3 or P3a). Importantly, the scoring of polysomnographic data showed that HR experienced more and longer awakenings during sleep than LR (16 min longer in average). These results suggest higher brain reactivity in HR than in LR facilitating nocturnal awakenings in HR. Indeed, Bastuji et al. (2008) showed that during sleep (N2 and REM sleep), an increased amplitude of the P3-like wave that was recorded in response to painful stimulation was strongly associated with subsequent arousal and awakening reactions. Interestingly, according to the “arousal—retrieval” hypothesis of Koulack and Goodenough (1976), such increase in intra-sleep wakefulness in HR could explain their high DRF. Indeed, according to Koulack and Goodenough, the sleeping brain is unable to encode new information in long-term memory and the transfer from short-term memory to long-term memory would require an awakening. Hence, in this framework, our ERPs results suggest that increased intra-sleep awakenings in HR would enable the encoding of the dream in long-term memory and then its recall at awakening in the morning. This ERPs study demonstrated for the first time differences in the brain reactivity of HR and LR. A great originality of the study was to show cerebral differences between HR and LR, not only during sleep but also during wakefulness. Importantly we showed that HR and LR differ in late brain responses associated with higher-level processes and notably automatic attention orienting. As a whole, this study suggests differences in the brain functional organization of HR and LR and supports the hypothesis of neurophysiological trait differences between subjects with high and low DRF.

In order to pursue the investigation of the neurophysiological differences between HR and LR we re-analysed the data of this EEG study (Eichenlaub et al., 2013) to investigate the oscillatory activity induced by complex sounds (first names) during sleep and wakefulness. We focused our analysis on the 8–12 Hz frequency band (alpha) for the following reasons.

In the sleep literature, the presence of alpha rhythm in the EEG is one of the criteria signaling the wakefulness state (Rechtschaffen and Kales, 1968; Silber et al., 2007). More precisely, according to the criteria published by the American Sleep Disorders Association (ASDA, 1992), an EEG arousal is an abrupt shift in EEG frequency during sleep, longer than 3 s, which may include theta, alpha and/or frequencies greater than 16 Hz but no spindle.

In the wakefulness literature, ongoing alpha rhythms were first discovered by Berger (1929) when the subject was awake, with eyes closed. This result raised two hypotheses: (1) alpha would operate as an idling rhythm when alertness decreases, or (2) alpha would be related to processing of visual information. Moosmann et al. (2003) showed later that ongoing alpha rhythms decrease at eyes opening even in the dark. This result supports the first hypothesis i.e., that alpha decrease would rather be induced by increased alertness than by stimulus appearance. Later studies then suggested that alpha decrease would also be related to the processing of stimuli (Holz et al., 2012).

In the visual modality, decreases in the power of alpha rhythms (also called event-related desynchronization) were observed over regions involved in the task realized by the subject. By contrast increases were found over regions irrelevant to the task (Klimesch et al., 2007). These results led authors to suggest that alpha rhythms would be involved in the active inhibition of the brain regions not involved in the current brain operations (Klimesch et al., 2007; Jensen and Mazaheri, 2010). Thus a decrease in alpha power in a specific brain region would correspond to a release of inhibition and an increased excitability.

In the auditory modality, few EEG studies investigated alpha oscillations. Taken together, they brought evidence that sounds induce an alpha decrease with a parietal topography (Yordanova et al., 2001; Mazaheri and Picton, 2005; Shahin et al., 2009), whereas the topography of alpha decrease induced by visual stimuli is more occipital (Mazaheri and Picton, 2005). Moreover, the more complex or task-relevant the stimuli, the greater and longer the alpha decrease, reflecting increased stimulus processing (Yordanova et al., 2001; Mazaheri and Picton, 2005; Shahin et al., 2009; Tavabi et al., 2011; Weisz et al., 2011). As a whole, results in the auditory modality are in agreement with those obtained in the visual modality, i.e., in both modalities a decrease in alpha power is observed after stimulus presentation.

Therefore, during wakefulness, alpha rhythms seem to play an important inhibitory role involved in the selection of stimuli to be processed and brain regions to be activated (or not inhibited) (Klimesch et al., 2007; Jensen and Mazaheri, 2010; Holz et al., 2012). Particularly, the alpha decrease seems to be related to the strength of stimulus processing.

Thus, in light of the functional role attributed to the alpha band modulation during sleep and wakefulness, and according to our ERP results one may expect that auditory stimuli induce different pattern of alpha activity in HR and LR. However, few studies investigated alpha activity in HR and LR during sleep (Goodenough et al., 1959; Lewis et al., 1966; Dumermuth et al., 1983; Benca et al., 1999; Cantero et al., 2002), and to our knowledge no study investigated alpha activity induced by stimulations during sleep and wakefulness in HR and LR.

In the present study, we tried to fill in this gap. We investigated alpha oscillatory activity in response to complex sounds (first names) during wakefulness and sleep in HR and LR. We used time-frequency analysis to investigate oscillatory power in the frequency-band 8–12 Hz [we ran an oscillatory analysis on the EEG data previously used for ERPs analysis in Eichenlaub et al. (2013)]. The ERPs analysis of the data (Eichenlaub et al., 2013) suggested a greater cerebral reactivity in HR than in LR. Hence, following Klimesch's hypothesis of an inhibitory role of alpha rhythms (Klimesch et al., 2007), one may expect a stronger decrease of alpha power in HR than in LR in response to first names during wakefulness. We also expected a difference between HR and LR during sleep but the direction of the effect could not be predicted due to the paucity of related results in the literature.

## Materials and methods

### Subjects and ethics statement

Approximately 1000 persons interested in participating in this study filled out a questionnaire concerning sleep and dreaming habits (the subjects were unaware that DRF was a criterion for subject selection). Subsequently, the subjects were contacted by telephone and asked “on average, how many mornings per week do you wake up with a dream in mind?”. A dream was previously defined as a long and bizarre story, an image that vanishes rapidly, or a feeling of having dreamt. Subjects were selected as High-recallers upon confirming dream recall (long stories or images) on more than three mornings per week, and as Low-recallers upon confirming dream recall (long stories, images or even a feeling of having dreamt) on less than two mornings per month. Eighteen High-recallers (mean DRF = 4.42 ± 0.25 SEM, dream recalls per week) and 18 Low-recallers (mean DRF = 0.25 ± 0.02) were selected. The following parameters did not differ between the groups: gender, age, habitual sleep duration, habitual sleep time, education level and the size of the place of residence (Schredl, 2008; Schredl and Reinhard, 2008; see Eichenlaub et al., 2013). The local ethics committee (Centre Leon Bérard, Lyon) approved this study, and subjects provided written, informed consent in conformity with the Declaration of Helsinki. The subjects were paid for their participation.

### Stimuli

The auditory stimuli were spectrally rich tones with a main frequency of 800 Hz and two harmonic partials (1600 and 3200 Hz), the subject's own first name (OWN) and an unfamiliar first name (OTHER). First names were digitally recorded by a neutral masculine voice using Adobe Audition 1.5 (Adobe software). After recording, maximum amplitudes of all stimuli were normalized. The mean durations of OWN (581 ms ± 86) and OTHER (598 ms ± 78) were not significantly different (Eichenlaub et al., 2012, 2013). Note that alpha responses were analyzed only for first-names in the present study. Responses to tones ERPs were presented elsewhere (Eichenlaub et al., 2013).

### Experimental design

The presentation of the four types of auditory stimuli obeyed the rules of a novelty oddball paradigm. Tones lasting 75 and 30 ms (including 5 ms rise/fall times) were used respectively as Standards (*p* = 0.82) and Deviants (*p* = 0.14). First names were presented as novel stimuli and the probability of occurrence was 0.02 for each of them. The stimuli were presented in a pseudo-randomized order so that (1) each Deviant followed at least two Standards, and (2) each Novel followed at least ten Standards and/or Deviants. Stimulus onset asynchrony (SOA) was set at 650 ms, except for the Standard following a Novel, which appeared 1260 ms after the Novel onset, whatever the duration of the Novel (Eichenlaub et al., 2012, 2013).

### Procedure

Subjects arrived in the lab at 7.00 p.m. after they had eaten. During ~1 h and a half, electrodes were fixed on their head and face. The subjects selected a movie among a choice of comedy or action movies. Then, subjects were installed in an acoustically dampened and electrically shielded room, earphones were inserted in their ears, and their hearing threshold was assessed using standard stimuli. The evening recording session started at 10.24 p.m. ± 45 min (duration, 1 h and 6 ± 9 min). Stimuli (around 120 Novels were presented in average) were presented binaurally at 50 dB above the subject's hearing level using the Presentation software (Neurobehavioral Systems). Subjects were instructed to watch the movie (silenced with subtitles) and to ignore the auditory stimuli (Eichenlaub et al., 2012). During the night session, stimuli were continuously presented (around 930 first names were presented in average), as subjects were in bed (Eichenlaub et al., 2013).

### Electrophysiological recordings

Twenty-one Ag/AgCl scalp electrodes were manually positioned according to the extended International 10–20 System (Fz, Cz, Pz; FP1, F3, FC1, C3, T3, CP1, P3, M1, O1 and their counterparts on the right hemiscalp). This relatively small number of electrodes was both compatible with sleep recording and with the use of scalp potential (SP) maps. We concentrated the electrodes around the sites expected to show between first name effects i.e., central and parietal sites. Contact between skin and electrodes was made using EC2 electrode cream Pactronic (Grass Product Group) and electrodes were fixed on the scalp using the paste TENSIVE (Parker Laboratories, Inc.). The reference electrode was placed on the tip of the nose, and the ground electrode on the forehead. The electro-oculogram (EOG) was recorded from 2 electrodes placed on the supraorbital ridge of the left eye and on the infraorbital ridge of the right eye. Muscle activity (EMG) was recorded from 2 electrodes attached to the chin. Electrode impedance was kept below 5 kΩ. The electrophysiological data (EEG, EOG, and EMG) were continuously recorded via a BrainAmp system (Brain Products GmbH, Germany) with an amplification gain of 12,500, a high-pass filter of 0.1 Hz and a sampling rate of 1000 Hz with an anti-aliasing low-pass filter (Eichenlaub et al., 2012, 2013).

### Sleep stage scoring

Sleep stages were scored off-line, visually by JBE according to standard criteria (Silber et al., 2007), and automatically by ASEEGA software (http://aseegaonline.com/pub/index.html) (Berthomier et al., 2007) to derive hypnograms based on 30 s epochs and to determine the vigilance state (wake, rapid eyes movements sleep—REM sleep, sleep stage 1—N1, sleep stage 2—N2 and slow wave sleep—N3) that occurred for every stimulus delivered during sleep. Only the sleep periods for which JBE and ASEEGA scores agreed were considered for analysis. The percentage of agreement between JBE and ASEEGA respective scoring was 82.9% with a kappa coefficient of 0.762 (epoch-by-epoch comparison; epochs scored as artefacts were excluded from the statistical analysis, Eichenlaub et al., 2013).

### Time-frequency analysis

Analysis was focused on responses to first names (novel stimuli). Oscillatory activities induced by the novel stimuli were characterized in each vigilance stage, separately. Trials were automatically excluded from analysis when the overall electrophysiological signal amplitude exceeded 150 μV during wakefulness and 400 μV in REM sleep, N2 and N3, during the [−500; 1500 ms] time-window. We investigated oscillatory activities by means of wavelet decomposition, which provides a good compromise between time and frequency resolutions. We used complex Gaussian Morlet's wavelets (complex waves with a Gaussian shape in the time- and frequency- domains) with a ratio f/σ_f_ = 7, f being the central frequency of the wavelet and σ_f_ the standard deviation of the Gaussian envelop in the frequency domain (Tallon-Baudry and Bertrand, 1999). With these parameters, the 10 Hz wavelet shows a bandwidth of 2.9 Hz in the frequency domain and a duration of 220 ms in the time domain. Each single trial signal was transformed in the time-frequency (TF) domain by convolution with the complex Morlet's wavelets on the time-window from −1000 to 2000 ms around stimulus onset. Averaging these TF powers would result in a power estimate of both evoked (phase-locked to stimulus onset) and induced (jittering in latency) activities in the TF domain. To reduce the contribution of stimulus phase-locked responses, for each subject, we averaged evoked potentials on the same time-window, and we subtracted this average from each single trial before time-frequency transformation. Then, the time-frequency responses of such corrected single trials were grand averaged across trials for each subject to get an estimate of the induced oscillation in the TF domain with few or no contamination by evoked activities.

We computed the oscillation power on the [−500; 1500 ms] time-window around first names onset in each group (HR and LR). A baseline correction was applied by subtracting the mean power between −300 and −100 ms before stimuli onset, in each frequency band.

### Statistical analysis

As we were interested in modulations of alpha oscillations, we performed statistical analysis in the frequency-window from 8 to 12 Hz. Moreover, as the Standard after a novel stimulus occurred 1260 ms after its onset we performed statistics on the time-window from 0 to 1200 ms.

Alpha reactivity to the first names was detected in the time–frequency domain at each electrode with successive non-parametric signed-rank Wilcoxon tests comparing the mean power in the 8–12 Hz frequency band over 200 ms windows moving by step of 100 ms (from 0 to 1200 ms) with the pre-stimulus power (mean power over a window between −300 and −100 ms before stimulus onset). These time-parameters are justified by the 220 ms duration of the 10 Hz wavelet. We used the following strategy to correct for multiple comparisons. In the temporal dimension we applied the Bonferroni correction (Abdi, 2007) and set the significance threshold at *p* < 0.005 (we divided 0.05 by 10 which is the number of tested time windows). In the spatial dimension, we considered an effect as significant only if it was detected on at least 2 adjacent electrodes (Caclin et al., 2008; Bidet-Caulet et al., 2012).

To compare alpha power induced in high and low recallers, non-parametric Mann–Whitney tests were performed at the electrodes showing a significant alpha reactivity to the first names. Mann–Whitney tests were performed on the mean power in the 8–12 Hz frequency band over 200 ms windows moving by step of 100 ms (from 0 to 1200 ms).

All analyses were performed with the ELAN Pack software developed at the Brain Dynamics and Cognition Team of the Lyon Neuroscience Research Center, Lyon, France (http://elan.lyon.inserm.fr) (Aguera et al., 2011).

## Results

### Behavioural results

Despite the uncomfortable nature of the experimental setup, sleep quality was generally preserved. For both groups, all sleep parameters evaluated were in the normal range. The sleep parameters did not differ between HR and LR with the exception of the duration of intra-sleep wakefulness (computed through the number of epochs scored as wakefulness during the sleep period; this measure did not include arousals or micro-arousals lasting less than 15 s). HR demonstrated longer intra-sleep wakefulness than LR (~15 min more on average). The number of awakenings (the number of phases composed of consecutive pages of awakening) was not significantly different between the two groups (HR, 17.5 ± 8.7; LR, 12.1 ± 11.9; *t*-test, *p* = 0.14), but the mean duration of the awakenings was (HR, 1.90 min ± 0.91; LR, 0.95 min ± 0.40; *t*-test, *p* < 0.005).

The dream reports obtained immediately after awakening in the morning confirmed a large DRF difference between the two groups. Although the subjects were in most cases awakened during REM sleep, only 33% of Low-recallers reported a dream while 94% of High-recallers did (see Eichenlaub et al., 2013).

### Time-frequency analysis

The average number of accepted trials (first names) was 127 ± 39 and 130 ± 39 during wakefulness in HR and LR respectively, 183 ± 52 and 214 ± 65 during sleep stage N2, 135 ± 32 and 133 ± 37 during sleep stage N3 and 114 ± 34 and 125 ± 35 during REM sleep.

#### Reactivity of alpha rhythm to first names

***Wakefulness.*** Analyzing the data of all subjects (high and low recallers), we found a significant decrease in alpha power in response to first names at centro-parietal sites (Pz and Cz, 500–800 ms; *p* < 0.005).

***REM sleep.*** During REM sleep, a large increase in alpha power was detected in response to first names at all electrodes, with a maximum over parietal electrodes. The increase of alpha power started at 100 ms post stimulus at centro-parietal electrodes and at 300 ms on the remaining electrodes (*p* < 0.005).

#### Comparison of alpha activity induced by first names in high and low recallers

**Figure 1:**
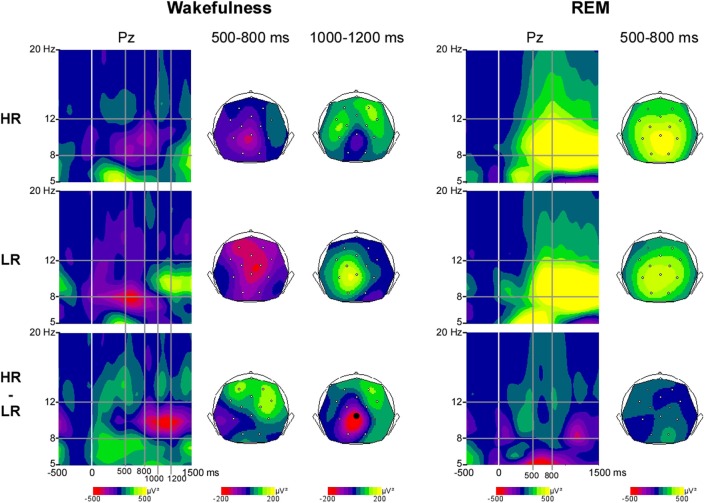
**Alpha power induced by first names during wakefulness and REM sleep in High-recallers and Low-recallers**. Time-Frequency (TF) plots for first names in HR, LR and HR minus LR, during wakefulness and REM sleep, at the electrode Pz, after baseline correction (baseline defined from −300 to −100 ms). x-axis: time, y-axis: frequency, the vertical white line indicates stimulus onset. Scalp topographies (back views) of alpha power are presented in the 500–800 ms time-window during wakefulness and REM sleep, and in the 1000–1200 ms time-window during wakefulness. The color scale represents in red (negative values) a decrease in oscillatory power, and in yellow (positive values) an increase. The electrode indicated by a black dot indicates electrode Pz where the difference between HR and LR reaches significance.

**Figure 2:**
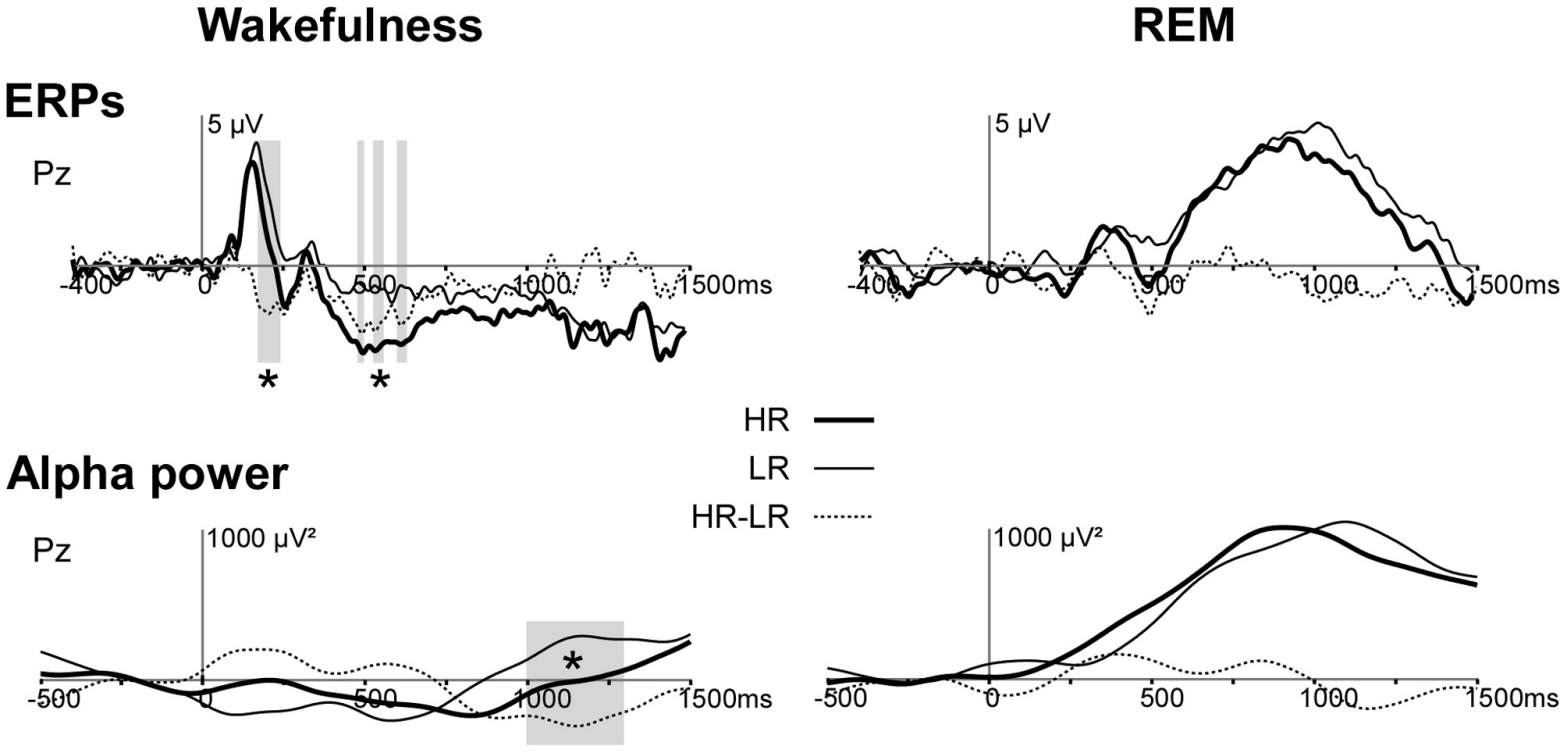
**Responses (ERPs and alpha power) to first-names in HR and LR during wakefulness and REM sleep at electrode Pz. Upper panel**. ERPs to first names (Eichenlaub et al., 2013). **Lower panel**. Time profiles of TF power in the 8–12 Hz frequency band for HR (bold continuous line), LR (thin continuous line) and HR minus LR (dotted line) in the wakefulness (left) and in the REM sleep condition (right). The gray-colored area indicates the time-window with a significant difference between HR and LR. HR, High-recallers, LR, Low-recallers. ^*^p < 0.05.

***Wakefulness.*** We found a group difference in the alpha activity (8–12 Hz) induced by first names at Pz between 1000 and 1200 ms post stimulus during wakefulness (*p* < 0.05, Figure 1). The first names induced a more sustained alpha decrease in HR than in LR (Figures 1, 2).

***REM sleep.*** During REM sleep the alpha power induced by first names was not different between high and low recallers (Figures 1, 2).

No results are provided from NREM sleep due to the strong overlap of the spectrum of K complexes, spindles, slow waves with the alpha band (8–12 Hz). As a consequence the genuine alpha reactivity to first names could not be correctly identified.

## Discussion

The present study aimed at better understanding the neurophysiological differences between HR and LR. In 18 High-recallers and 18 Low-recallers, we investigated alpha oscillatory activity (8–12 Hz) in response to first names presented as auditory novel stimuli in a stream of simple tones during sleep and wakefulness. First names were presented at a low frequency (4% of stimuli) in a novelty oddball paradigm while subjects were watching a silent movie with subtitles in the evening and during sleep at night. Our objective was to compare between HR and LR the reactivity of alpha rhythms to novel stimuli during wakefulness and sleep.

According to the dominant hypothesis, alpha rhythms would be involved in the active inhibition of the brain regions not involved in the current brain operations (reviews: Klimesch et al., 2007; Jensen and Mazaheri, 2010). A decrease in alpha power in a specific brain region would thus correspond to a release of inhibition and an increased excitability. According to this hypothesis, in our study, HR would present a longer release of inhibition than LR after stimuli presentation, suggesting a deeper processing of first names during wakefulness. This result extends our previous ERP results showing a more complex processing of stimuli in HR than in LR (Eichenlaub et al., 2013).

Contrary to our hypothesis we found no group difference in induced alpha power during REM sleep. One possibility to explain this negative result is that increases in alpha power in HR may have resulted in awakenings. Indeed, contrary to wakefulness, in REM sleep, stimulations induce an increase in alpha power and not a decrease. As alpha is a rhythm characteristic of the wake state, a strong increase in alpha power may destabilize REM sleep and induce a transition from sleep to wakefulness. If so, the stimuli showing a great alpha effect have been excluded from the sleep analysis because they pertained to a page showing signs of wakefulness. Our results support this hypothesis since HR experienced more intra sleep wakefulness than LR (30 ± 4 vs. 14 ± 5 min in HR vs. LR on average, see Eichenlaub et al., 2013). In addition, we observed that the delay between the pages scored as intra-sleep wakefulness and the last preceding first name was shorter in HR than in LR. Importantly, this delay was less than 15 s in both groups. Indeed, an awakening is considered stimulus-related if it occurs within 15 s after stimulus onset (Bastuji et al., 2008; Arzi et al., 2012). This result suggests that first names (which elicit larger ERPs in HR than in LR) are more arousing in HR than in LR. Taken together, ERP results suggest the possibility of a causal link between the amplitude of the ERPs to auditory stimuli during sleep and intra-sleep awakenings (possibly due to a stimulus-induced alpha power increase) (Figure 2). In this case, the higher brain reactivity in HR during both wakefulness and sleep would contribute to their higher frequency of dream report, by increasing intra-sleep wakefulness which would in turn facilitate the encoding of dreams in memory according to the “arousal-retrieval” model (Koulack and Goodenough, 1976).

### Implications for the understanding of the functional role of alpha rhythm

Two interpretations can be put forward to explain that alpha reactivity to stimuli has two opposite directions during wakefulness (decrease) and REM sleep (increase) (data analysis in all subjects, *N* = 36;).

Cantero et al. (2002) proposed that increased alpha power during REM sleep would reveal micro-arousal without awakening. They hypothesized that micro-arousals facilitate the momentary contact with the external environment without alterations in the REM continuity (Cantero et al., 2002). Some recent results argue in favor of this hypothesis. By systematically challenging sleep with realistic and varied acoustic disruption, McKinney et al. (2011) found that sleepers exhibited markedly greater sensitivity to sounds during moments of elevated alpha expression during non REM sleep. According to Cantero's hypothesis, the increase of alpha power induced by first names in our study could reveal their disturbing impact and could reflect a destabilization of sleep without awakening.

Yet, the functional role attributed to alpha rhythms in REM sleep by Cantero et al.'s hypothesis is not consistent with their functional role during wakefulness according to the inhibition-timing hypothesis (Klimesch et al., 2007; Jensen and Mazaheri, 2010). According to the wakefulness literature, an increase in alpha power would rather be associated with cortical inhibition than with cortical excitation. In agreement with this hypothesis, Benca et al. (1999) suggested that increased alpha power might represent cortical deactivation during sleep. If we interpret our results in line with the latter hypothesis, the increased alpha power in response to first names would be associated with an inhibition process resulting in a diminished processing of the first names. This reduced processing may result in a protection of sleep, preventing the subject from awakening. This hypothesis is compatible with the high auditory awakening threshold observed during REM sleep (auditory awakening threshold is higher in REM sleep as compared to sleep stage N2) (Rechtschaffen et al., 1966). Note that in our experiment, when the subjects were allowed to sleep they had already heard the auditory paradigm for 1 h. As a consequence, the first names may have been perceived much more as disturbing rather than as alerting stimuli. It is likely that in such circumstances the brain develops an inhibiting strategy in order to preserve sleep. However, we can infer that if first names, and especially the OWN, would have been uttered once during silent REM sleep, brain processes triggered by this stimulus might have been different and could have led to subject's awakening.

The comparison of the brain activity in HR and LR provides argument rather in favor to the hypothesis of an activating role of alpha rhythm during sleep. Indeed, our results showed increased reactivity in HR be it with ERPs during sleep and wakefulness, or with alpha reactivity during wakefulness. In addition intra-sleep wakefulness was longer in HR than in LR. All these results argue in favor of increased reactivity in HR as compared to LR. As a consequence one may interpret the absence of alpha difference between the 2 groups during REM sleep as a false negative. Indeed, the increased reactivity in HR resulted in increased post-stimulus ERPs amplitude. If it also induced a post-stimulus increase in alpha power, such increase may have destabilized sleep and may have led to awakenings. If true, awakening would systematically mask the increase in alpha power in HR since trials followed by awakening are excluded from the analysis of sleep.

### Implications for neuroscientific, psychological and philosophical theories of dreaming

Our results (ERPs and alpha) as well as previous ones (Solms, 1997; Palagini et al., 2004; Noreika et al., 2009) challenge the idea that investigating REM sleep is enough to understand the dreaming brain. By contrast, they are the first results to suggest that dream production and/or memorization is/are promoted by a specific functional organization of the brain. They also provide a possible neurophysiological substrate to the personality, creativity and sleep differences previously found between high and low recallers (Schredl et al., 2003). As such these results raise numerous questions. Can a subject evolve from low to high-recaller or from high to low-recaller? If so, which parameters are responsible for such evolution? Does the functional organization of his/her brain evolve concomitantly? And how? Does his/her personality evolve concurrently? Further studies will have to address these issues to progress in our understanding of the dreaming brain.

### Implications for the understanding of the relationship between dream production and dream recall

At the theoretical level, contrasting HR and LR cannot help to resolve the memory/production issue i.e., disentangle between the respective involvement of dream production and dream memorization in the ability to recall dreams. This paradigm may nonetheless help to progress providing some clues.

Our results showing intra-sleep wakefulness differences between HR and LR, strongly suggest that memory processes participate in the DRF difference between groups in the context of the arousal-retrieval model (Koulack and Goodenough, 1976). Our previous ERP results, on the other hand, showing brain reactivity differences during sleep between HR and LR (i.e. revealing different brain states) leave open the possibility that HR produce more dreams than LR. The results of our studies are thus in favor of mixed memory and production differences between HR and LR

Contrasting HR and LR with neuroimaging techniques (functional Magnetic Resonance Imaging—fMRI, or PET may also help to progress in the understanding of the relationship between dream production and dream recall. Indeed, identifying the brain regions showing the greatest activity difference between HR and LR during sleep may provide clues. If, for example, the brain regions differentiating the two groups are involved in memory processing (HR showing more activity than LR in the hippocampus during sleep for example), this would be an argument in favor of the hypothesis proposing that “no dream recall” result from no memory of the dream (encoding or recall) rather than from no production of the dream.

### Conflict of interest statement

The authors declare that the research was conducted in the absence of any commercial or financial relationships that could be construed as a potential conflict of interest.
